# Cytokine profiles in pregnant gilts experimentally infected with porcine reproductive and respiratory syndrome virus and relationships with viral load and fetal outcome

**DOI:** 10.1186/s13567-014-0113-8

**Published:** 2014-12-06

**Authors:** Andrea Ladinig, Joan K Lunney, Carlos JH Souza, Carolyn Ashley, Graham Plastow, John CS Harding

**Affiliations:** Department of Large Animal Clinical Sciences, Western College of Veterinary Medicine, University of Saskatchewan, Saskatoon, SK Canada; U.S. Department of Agriculture, Animal Parasitic Diseases Laboratory, Beltsville Agricultural Research Center, Agricultural Research Service, Beltsville, MD USA; EMBRAPA Pesca e Aquicultura, Palmas, TO Brazil; Department of Agricultural, Food, and Nutritional Science, Faculty of Agricultural, Life and Environmental Sciences, University of Alberta, Edmonton, AB Canada

## Abstract

In spite of extensive research, immunologic control mechanisms against Porcine Reproductive and Respiratory Syndrome virus (PRRSv) remain poorly understood. Cytokine responses have been exhaustively studied in nursery pigs and show contradictory results. Since no detailed reports on cytokine responses to PRRSv in pregnant females exist, the objectives of this study were to compare host cytokine responses between PRRSv-infected and non-infected pregnant gilts, and to investigate relationships between cytokine levels in infected gilts and viral load or fetal mortality rate. Serum samples and supernatants of peripheral blood mononuclear cells (PBMC) either stimulated with PRRSv or phorbol myristate acetate/Ionomycin (PMA/Iono) were analyzed for cytokines/chemokines: interleukins (IL) 1-beta (IL1β), IL4, IL8, IL10, IL12, chemokine ligand 2 (CCL2), interferon alpha (IFNα) and interferon gamma (IFNγ). Three cytokines (IFNα, CCL2, IFNγ) in gilt serum differed significantly in inoculated versus control gilts over time. In supernatants of PRRSv stimulated PBMC from PRRSv-infected gilts, levels of IFNα were significantly decreased, while IL8 secretion was significantly increased. PRRSv infection altered the secretion of all measured cytokines, with the exception of IFNα, from PBMC after mitogen stimulation, indicating a possible immunomodulatory effect of PRRSv. IFNα, CCL2, and IFNγ in serum, and IFNγ in supernatants of PMA/Iono stimulated PBMC were significantly associated with viral load in tissues, serum or both. However, only IFNα in supernatants of PRRSv stimulated PBMC was significantly associated with fetal mortality rate. We conclude that of the eight cytokines tested in this study IFNα was the best indicator of viral load and severity of reproductive PRRSv infection.

**Electronic supplementary material:**

The online version of this article (doi:10.1186/s13567-014-0113-8) contains supplementary material, which is available to authorized users.

## Introduction

Cytokines and chemokines play a key role in the regulation of the innate, humoral (T-helper 2 [Th2]) and cellular (T-helper 1 [Th1]) immune responses [1]. E*arly cytokines* including the type I interferons and pro-inflammatory cytokines (interleukins 1 (IL1), IL6 and tumor necrosis factor-alpha (TNFα)), and *late cytokines* such as interferon-gamma (IFNγ), are important regulators of adaptive immune responses [2]. Two important chemokines are interleukin 8 (IL8 or CXCL8), a potent recruiter of neutrophils to sites of infection, and chemokine ligand 2 (CCL2), which induces the migration of monocytes from blood to become tissue macrophages [3]. Antiviral or type I interferons are produced by a variety of cells, with plasmacytoid dendritic cells (pDC) or interferon producing cells (IPCs) being specialists in this task [3]. Type II interferon, IFNγ, and IL12 are key inducers of Th1 immune responses [2,3]. The functions of IL10 are diverse, but principally aimed at immune regulation [3,4]. Unlike in human or mouse, in which IL4 is the major Th2 cytokine [5-7], the role of IL4 in pigs is not completely clear and its expression in vivo following viral infection is usually low or undetectable [8-10].

Recently, bead-based multiplex assays, also known as Fluorescent Microsphere Immunoassays (FMIA), became available for measurement of cytokines in porcine specimens. FMIA allows high throughput, simultaneous detection and quantification of multiple analytes and significantly reduced time and sample volume requirements [11,12]. For detection of cytokines, FMIA technology relies on the availability of capture and detection antibodies (Abs) enabling specific and sensitive measurement of the respective analytes. Because a limited number of swine antibodies are available and not all work well in multiplex FMIA the use of FMIA to detect swine cytokines is presently limited [13].

Cytokine responses to Porcine Reproductive and Respiratory Syndrome virus (PRRSv) infection have been exhaustively studied using both in vivo and in vitro models. A thorough review is beyond the scope of the present paper. However, reports on cytokine responses to PRRSv infection in vivo contain contradictory results and were mainly performed in nursery pigs using respiratory models. Rowland et al. [14] used a reproductive model to investigate cytokine responses in PRRSv-infected fetuses but not in dams. To our knowledge, no detailed reports of cytokine responses to PRRSv infection in pregnant sows or gilts exist. Therefore, the objectives of the present study were: 1) to compare host cytokine responses between PRRSv-infected and non-infected gilts following experimental infection in the third trimester of gestation; 2) to investigate relationships between cytokine levels and viral load in gilt serum and gilt tissues; and 3) to investigate relationships between cytokine levels and fetal mortality rate defined at the level of the gilt as percent dead fetuses per litter. Three specific host responses were evaluated over 19 days post inoculation (dpi): 1) cytokine production in gilt serum, 2) cytokine production in supernatants of PRRSv-stimulated peripheral blood mononuclear cells (PBMC), and 3) cytokine production in supernatants of phorbol myristate acetate/Ionomycin (PMA/Iono) stimulated PBMC.

## Materials and methods

### Animal experiment and sample collection

The experimental infection protocol is described in detail in Ladinig et al. [15]. Briefly, on experimental day 0 (D0), 114 pregnant Landrace gilts (gestation day 85 ± 1 over 12 biweekly replicates) were inoculated with PRRSv isolate NVSL 97–7895 (1 × 10^5^ TCID_50_; 2 mL intramuscular and 1 mL into each nostril) (INOC), while 19 control gilts were similarly sham inoculated (CTRL). PRRSv negativity of gilts was confirmed before delivery to the University of Saskatchewan by ELISA (IDEXX PRRS X3 Ab test, IDEXX laboratories, Inc., Maine, USA) and PCR (Tetracore PRRS real-time PCR kit, Tetracore, Inc., Rockville, USA). Furthermore, all gilts were negative for PRRSv RNA by a strain-specific in-house quantitative reverse transcription polymerase chain reaction (qRT-PCR) in serum on D0 [15]. Serum and whole blood samples were collected on D0, D2, D6, D19 and D21. On D21 (gestation day 106 ± 1), gilts were humanely euthanized and necropsy examinations were performed on gilts and their fetuses. Fetal preservation status was recorded and the percent dead fetuses were calculated for each litter. PRRSv RNA concentrations were measured in gilt serum and gilt tissue samples (lung, tonsil, reproductive and tracheobronchial lymph node) collected at termination using qRT-PCR [15]. The experiment was approved by the University of Saskatchewan’s Animal Research Ethics Board, and adhered to the Canadian Council on Animal Care guidelines for humane animal use (permit #20110102).

### PBMC stimulation

PBMC were isolated from whole blood collected on D0, D2, D6 and D19 by gradient centrifugation using lymphocyte separation medium (Ficoll-Paque™ PLUS, GE Healthcare). Cells were re-suspended in RPMI medium 1640 (RPMI) (Life Technologies, Burlington, ON, Canada) supplemented with antibiotics (1% Pen/Strep) and 10% fetal bovine serum (FBS) (Life Technologies, Burlington, ON, Canada). On the day of isolation, freshly isolated PBMC were seeded in 48-well tissues culture plates (1 × 10^6^ cells/ well, total volume 500 μL). Duplicate wells for each stimulation time point were stimulated with either 10 ng/mL PMA (Sigma-Aldrich, Oakville, ON, Canada) and 250 ng/mL ionomycin (Sigma-Aldrich, Oakville, ON, Canada) (PMA/Iono) or with PRRSv isolate NVSL 97–7895 (multiplicity of infection =1). Unstimulated cells were seeded in duplicate wells containing cell culture medium only. Plates were incubated at 37 °C in 5% CO_2_ for either 36 h (PMA/Iono stimulation) or 60 h (PRRSv stimulation). Cells and supernatants from duplicate wells were pooled and centrifuged (400 *g*, 10 min). Supernatant aliquots were stored frozen at −80 °C until further testing.

### Cytokine testing

Serum samples (D0, D2, D6, D21) and PBMC supernatants (D0, D2, D6, D19) were analysed for the following innate, regulatory, Th1 and Th2 cytokines/chemokines by FMIA: IL1β, IL8, CCL2, IFNα, IL10, IL12, and IL4. Due to the lack of availability of antibodies suitable for FMIA, an enzyme-linked immunosorbent assay (ELISA) was used to measure IFNγ.

### Multiplex fluorescent microsphere immunoassay (FMIA)

**Figure 1:**
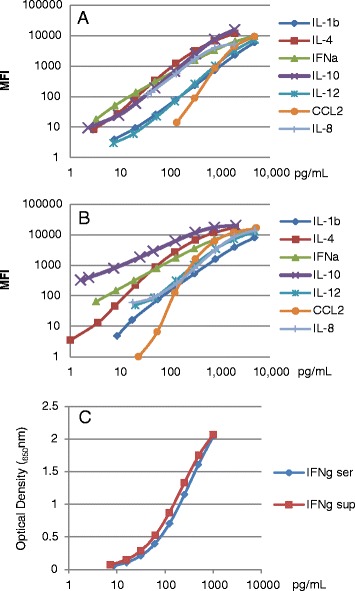
**Examples of standard curves obtained by FMIA and ELISA.** Examples of standard curves of recombinant protein standards serially diluted in serum **(A)** or cell culture medium **(B)** for the seven cytokines tested by FMIA; **(C)** examples of IFNγ-standard curves (recombinant protein serially diluted) in serum (ser) or cell culture medium (sup) by ELISA.

For serum samples, a 7-plex in-house FMIA assay was developed as previously described with several modifications [12]. Beads, standards and Ab pairs used for the different cytokines are listed in Table 1. Briefly, capture antibodies were covalently coupled to magnetic beads (Biorad, Mississauga, ON, Canada). Coupling efficiency was tested by staining newly coupled beads with goat anti-mouse IgG-Phycoerythrin labeled Ab (Life Technologies, Burlington, ON, Canada) and comparing fluorescence intensity of the newly coupled beads with existing batches (generously provided by Dr C. Souza, USDA, Beltsville, MD, USA). Serum from a healthy, non-infected gilt with no measurable levels of relevant cytokines by FMIA was used to prepare serial dilutions of cytokine standards (Figure 1A). The same serum was used as negative control on each FMIA plate. All serum samples were tested at a 1:3 dilution in phosphate buffered saline (PBS, pH 7.4) supplemented with 1% bovine serum albumin (BSA) and 0.05% sodium azide (PBS-BN). Samples and standards were incubated with magnetic beads in duplicate wells of a 96-well plate for 2 h. All incubations were performed in the dark (aluminum foil cover) at room temperature on a rotating plate shaker (500 rpm). A 96-well plate washer (Bio-Plex Pro™ II Wash Station, Biorad, Mississauga, ON, Canada) was used to perform 3 washes with PBS +0.05% Tween 20 (PBST) after each incubation step. Fifty μL of anti-cytokine, biotinylated, secondary antibody (Ab) diluted in PBS-BN were added to each well and plates were incubated for 90 min. The optimal concentration of each biotinylated detection Ab was determined by titration. Finally, beads in each well were incubated in 50 μL of a solution containing 10 ug/mL of Strepavidin-R-Phycoerythrin (SAPE, PJRS20 Prozyme, Hayward, CA, USA) for 30 min, washed, and re-suspended in 100 μL PBST. Coupled microspheres were analyzed on a Bio-Plex® 200 system (Biorad, Mississauga, ON, Canada) and analyzed with the Bio-Plex Manager software version 6.1 (Biorad, Mississauga, ON, Canada). All mean fluorescence intensity (MFI) measurements were background corrected by subtracting the MFI of the negative control (control serum diluted 1:3 in PBS-BN) from the MFI for the relevant analyte in each sample.

**Table 1:** **Standards, capture and detection antibodies used by FMIA and ELISA**

**Cytokine (bead region)**	**Standard (source)**	**Capture Ab (source)**	**Detection Ab (source)**
IL1β (26)	DY681, part 841042 (RD)	DY681, part 841040 (RD)	BAF 681 (RD)
IL8 (27)	SD061 (L)	MCA1660 (C)	MAB5351 (RD)
CCL2 (53)	RP0017S-025 (C)	Anti-poCCL2 clone 5–2 (Lu)	Anti-poCCL2 clone 18–1 (Lu)
IFNα (45)	17105-1 (C)	GTX11408 (GT)	27105-1 (C)
IL10 (28)	CSC0103, part SD064 (L)	ASC0104 (L)	ASC9109 (L)
IL12 (36)	DY912, part 841099 (RD)	MA0413S-500 (C)	BAM9122 (RD)
IL4 (34)	CSC1283, part 5S.128.10 (L)	CSC1283, part 5S.128.09 (L)	ASC0849 (L)
IFNγ (ELISA)	CSC4033, part SD066 (L)	CSC4033, part ASC4934D (L)	CSC4033, part ASC4839D (L)

(RD) R&D Systems, Minneapolis, MN, USA; (L) Life technologies, Burlington, ON, Canada; (C) Cedarlane, Burlington, ON, Canada; (GT) GeneTex, Irvine, CA, USA; (Lu) Lunney lab, Beltsville, MD, USA (Lunney et al., manuscript in preparation).

PBMC supernatants were tested in a similar manner. However, due to high cytokine concentrations present after either PRRSv or PMA/Iono stimulation, a higher sample dilution was required for certain analytes and use of a 7-plex assay was therefore not feasible. For supernatants of PRRSv stimulated PBMC, CCL2 was analyzed as a single-plex assay at a 1:100 dilution, while the remaining analytes were tested in a 6-plex assay at a 1:5 dilution. For supernatants of PMA/Iono stimulated PBMC, IL-8 was analyzed at a 1:20 dilution, while the remaining analytes were tested in a 6-plex assay at a 1:5 dilution. Cell culture medium (RPMI +10% FBS +1% Pen/Strep) at the relevant dilution in PBS-BN was used as negative control and to prepare standard curves (Figure 1B). Biotinylated detection Abs were titrated in order to determine the optimal concentration to use in testing PBMC supernatants. Results from supernatants of PRRSv or PMA/Iono stimulated PBMC were adjusted by subtracting the cytokine levels in supernatants of unstimulated cells cultured for the same time point (36 or 60 h). These are reported as “adjusted” cytokine values herein.

### IFN-γ ELISA

The assay was carried out using the Novex Swine IFNγ antibody duoset kit (Life Technologies Inc., Burlington, ON, Canada) according to the manufacturer’s instructions. Low coefficient of variation (CV) Immulon 4 HBX 96 well plates (VWR International, Mississauga, ON, Canada) were coated with capture Ab diluted in Coating Buffer B (Life Technologies Inc., Burlington, ON, Canada). 5X Assay Buffer (Novex Antibody Pair Buffer Set, Life Technologies Inc., Burlington, ON, Canada) diluted in ddH_2_O was used as a blocking solution and for dilution of detection and Streptavidin-HRP Abs and samples as appropriate. Plates were washed with PBST using a Bio-Plex Pro II Wash Station. Peroxidase-labeled conjugates were detected by the addition of SureBlue Reserve TMB Microwell Substrate (Mandel Scientific Company Inc, Guelph, ON, Canada). Color was developed in the dark until the optical density of the highest standard reached approximately 2.0 at a wavelength of 650 nm without addition of Stop Solution. Plates were read in a Vmax Microplate Reader (Molecular Devices Corporation, Downingtown, PA, USA) using SoftMaxPro software Version 1.1 (Sunnyvale, CA, USA). The standard curve (1000 pg/mL to 8 pg/mL) was constructed using doubling dilutions (Figure 1C). Concentrations of IFNγ in samples were estimated by comparison with a 4-parameter standard curve generated by the software. Serum samples were diluted 1:3 in assay buffer whereas the standard diluent was a 1:3 solution of control gilt serum in assay buffer. Supernatants of PRRSv and PMA/Iono stimulated PBMC were tested at 1:3 and 1:100 dilutions, respectively, in assay buffer. The standard diluent was a corresponding dilution of cell culture medium in assay buffer. As above, IFNγ levels in PBMC supernatants were adjusted by subtracting the IFNγ levels in supernatants of unstimulated cells; adjusted IFNγ values are compared and reported herein.

### Cytokine quality control (QC)

Protein standards (Table 1) were used to prepare high and low concentration quality controls in either control serum or cell culture media at the relevant dilution in PBS-BN. Both high and low quality controls were run in duplicate on each FMIA and ELISA plate. Process behavior control charts were used to monitor the inter-plate variation of each cytokine concentration in the QC samples, as well as the MFI of the highest and lowest standard on each FMIA plate. Plates were repeated if values fluctuated above or below the 3-sigma natural process limits on the appropriate control charts. Individual samples were repeated if the replicate CV was >20% as calculated by the instrument’s software.

### Statistical analysis

Separate statistical analyses were conducted using Stata 13 (STATA Corp, College Station, Texas, USA) to address each of three objectives. To meet the key model assumptions, data were transformed (logarithm base_10_ or zero-skewness (lnskew0 function in STATA)) as appropriate. First, to determine if cytokine levels differed between INOC and CTRL gilts, multilevel mixed-effects linear regression models were developed. These models used gilt as a random effect and accounted for repeated measures by day. All remaining analyses used data from INOC gilts only and specifically focused on the cytokines that differed statistically between INOC and CTRL gilts. Our second objective was to determine potential relationships between cytokine levels (serum and supernatants) and PRRS viral load in serum and tissues. For these analyses, multilevel mixed-effects linear regression models controlling for experimental replicate were used. The area under the curve (AUC) for PRRSv RNA (target copies/μL, D0-D21) and cytokine protein (pg/mL) in serum (D0-D21) and supernatants (D0-D19) over time was calculated using the formula AUC = (t_1_-t_0_)(a_1_ + a_0_)/2 + (t_2_-t_1_)(a_1_ + a_2_)/2 + … + (t_n_-t_n-1_)(a_n-1_ + a_n_)/2. Our third objective was to determine potential associations between cytokine levels in serum (AUC) and fetal survival rate (percentage of dead fetuses per litter). For all analyses, cytokine levels in sera or adjusted cytokine levels in supernatants (as described above) were used.

To account for multiple comparisons, all associations were considered statistically significant if *P* < 0.01. All final models were evaluated to ensure normality and homoscedasticity of residuals.

## Results

### Cytokine differences between inoculated and control gilts

Examples of standard curves of recombinant protein standards serially diluted in either serum or cell culture medium for the seven cytokines tested by FMIA are presented in Figures 1A and 1B. Examples of standard curves for the IFNγ ELISA are presented in Figure 1C.

**Figure 2:**
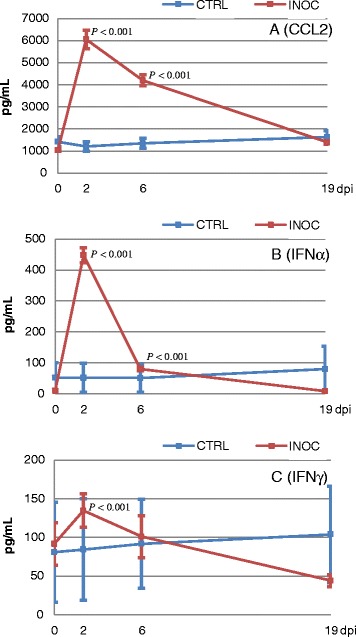
**CCL2, IFNα and IFNγ levels in gilt serum over time.** Mean (± SEM) CCL2 **(A)**, IFNα **(B)** and IFNγ **(C)** levels in serum from 111 INOC and 19 CTRL gilts across respective study days; *P*-values indicate significant differences between INOC and CTRL gilts on individual days.

Cytokine levels showed a high degree of variation between individual gilts in all sample types (Additional file 1, Additional file 2 and Additional file 3). Serum levels of CCL2, IFNα and IFNγ differed significantly between INOC and CTRL gilts over time (*P* < 0.001 for all). INOC gilts showed a significant increase in CCL2 and IFNα in sera collected on D2 and D6, whereas IFNγ was increased significantly on D2 only (Figure 2). IFNγ levels measured in serum of INOC gilts on D21 were lower than CTRL gilts; however, differences were not statistically significant. High serum levels of IL1β and IL8 at all 4 time points were measured in individual INOC and CTRL gilts, but PRRSv inoculation had no effect on these analytes over time (Additional file 1). Levels of IL12, IL10 and IL4 were very low in serum of both INOC and CTRL gilts and did not significantly differ over time (Additional file 1).

**Figure 3:**
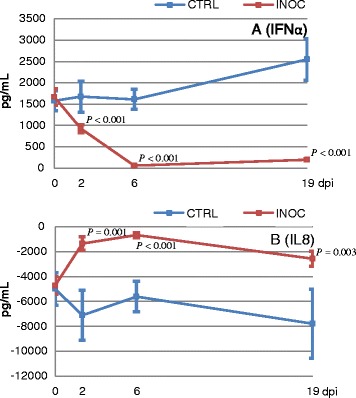
**Adjusted IFNα and IL8 levels in supernatants of PRRSv stimulated PBMC over time.** Mean (± SEM) adjusted IFNα **(A)** and IL8 **(B)** levels in supernatants of PRRSv stimulated PBMC from 111 INOC and 19 CTRL gilts across respective study days. *P*-values indicate significant differences between INOC and CTRL gilts on individual days. Adjusted cytokine levels were calculated by subtracting values in supernatants of unstimulated cells from PRRSv stimulated cells.

In supernatants of PRRSv stimulated PBMC, levels of IFNα and IL8 differed significantly between INOC and CTRL gilts over time (*P* ≤ 0.001). PRRSv stimulation induced high levels of IFNα in PBMC from CTRL gilts and from INOC gilts before inoculation. Levels of IFNα were highly variable, ranging from 62 to 17025 pg/mL. A significant reduction in IFNα was detected in stimulated PBMC from INOC gilts compared to CTRL on D2, D6, and D19 (Figure 3A). Decreased levels were detected in all INOC gilts but to varying degrees. IL8 levels in supernatants of PRRSv stimulated PBMC were significantly increased on D2, D6, and D19 in INOC versus CTRL gilts (Figure 3B). Levels of IL1β, CCL2, IFNγ, IL4, IL10 and IL12 in supernatants of PRRSv stimulated PBMC were either not detectable or low, and did not significantly differ between INOC and CTRL gilts over time (Additional file 2).

**Figure 4:**
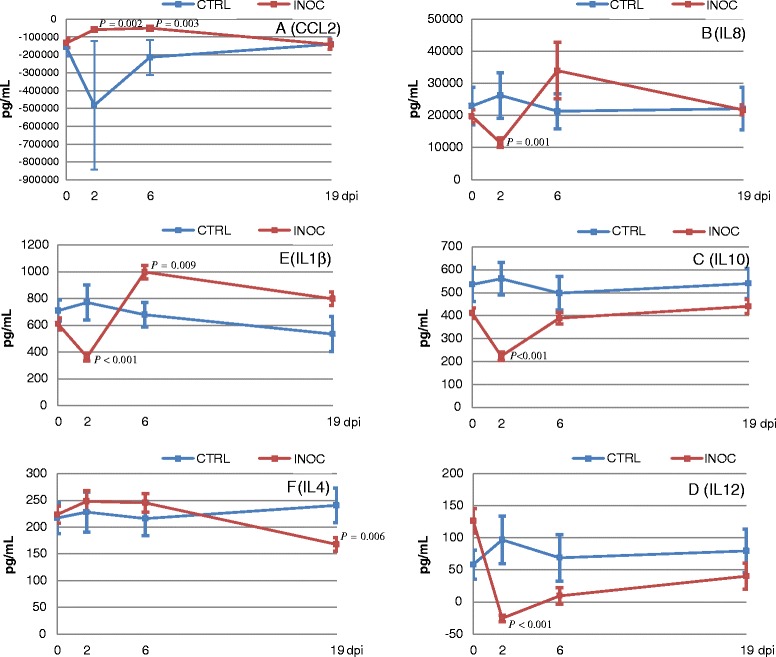
**Adjusted CCL2, IL8, IL10, IL12, IL1β, and IL4 levels in supernatants of PMA/Iono stimulated PBMC over time.** Mean (± SEM) adjusted CCL2 **(A)**, IL8 **(B)**, IL10 **(C)**, IL12 **(D)**, IL1β **(E)**, and IL4 **(F)** levels in supernatants of PMA/Iono stimulated PBMC from 111 INOC and 19 CTRL gilts across respective study days. *P*-values indicate significant differences between INOC and CTRL gilts on individual days. Adjusted cytokine levels were calculated by subtracting values in supernatants of unstimulated cells from PMA/Iono stimulated cells.

IFNα was not induced by PMA/Iono stimulation. All other cytokines from PMA/Iono stimulated PBMC differed between INOC and CTRL gilts over time (*P* < 0.001 for all except IFNγ where only a trend was reported (*P* = 0.039)) (Additional file 3). Among the 7 cytokines measured, four discrete patterns were observed reflecting the potential effects of gilt PRRSv infection on PBMC PMA/Iono responsiveness. First, PRRSv infection resulted in a significantly increased CCL2 response to PMA/Iono stimulation on D2 and D6 but not on D19 (early induction; Figure 4A). These statistical differences however, are the result of a single outlier CTRL gilt with massive spontaneous production of CCL2 from unstimulated PBMC on day 2. With this outlier removed, CCL2 production significantly differed between INOC and CTRL over time (*P* = 0.01) with levels in INOC increased on D2 and D6 compared to CTRL. After adjusting for multiple comparison this was considered a trend (D2 *P* = 0.027, D6 *P* = 0.019). By contrast, levels of IL8, IL10, and IL12 were significantly decreased on D2 in INOC compared to CTRL gilts (early suppression; Figures 4B-D). Following PMA/Iono stimulation, levels of IL1β decreased similarly on D2, but were significantly increased in PBMC from INOC versus CTRL gilts on D6 (early suppression with rebound; Figure 4E). Finally, IL4 demonstrated late suppression with levels from PMA/Iono stimulated PBMC significantly decreased in INOC versus CTRL on D19. Prior to D19, IL4 levels numerically increased in INOC gilts (Figure 4F). Similarly, IFNγ levels decreased in INOC gilts on D19; however, differences were not significant but rather a trend (*P* = 0.023; Additional file 3).

### Relationships between cytokines levels and PRRS viral load

**Figure 5:**
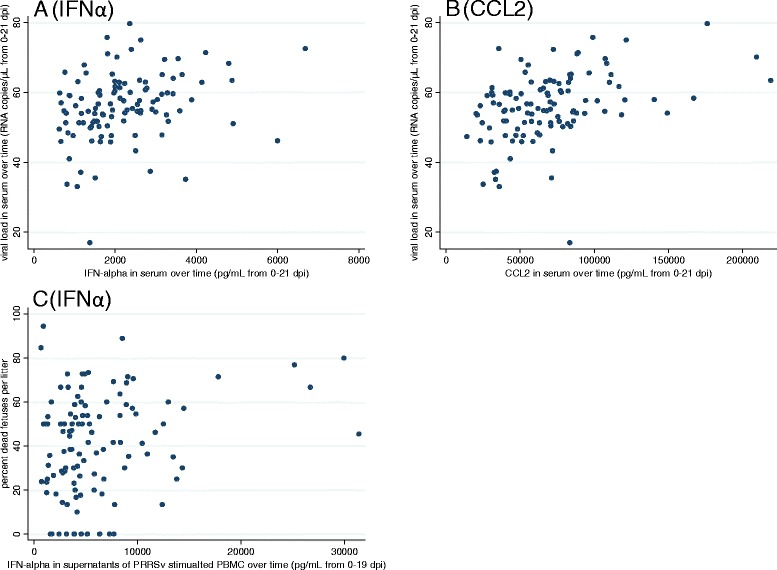
**Scatter plots of selected cytokines in serum and supernatants of PRRSv stimulated PBMC versus viral load in gilt serum or percent dead fetuses per litter.** The levels of IFNα **(A)** and CCL2 **(B)** in serum over time are plotted against the viral load in gilt serum over time (area under the curve (AUC) from 0 to 21 dpi) illustrating individual variation. **(C)** The levels of IFNα in supernatants of PRRSv stimulated PBMC over time (AUC) are plotted against the percentage of dead fetuses per litter. Animals in the upper right quadrant are helping to drive the relationship. No gilts had high cytokine levels and low viral load in serum or low percent of dead fetuses per litter.

Detailed results on viral loads in gilt serum and tissues can be found in Ladinig et al. [15]. Briefly, all INOC gilts were viremic on D2 and D6, and 94/111 (84.7%) remained viremic until termination. The percentage of tissues that tested positive by PRRSv qRT-PCR at termination (D21) from INOC gilts were 90.1 (lung), 99.1 (tonsil), 100 (reproductive lymph node), and 99.1 (tracheobronchial lymph node), respectively. The mean viral loads of positive samples were 3.5 (±1.2) log_10_ RNA copies/mg in lung, 5.6 (±0.8) log_10_ RNA copies/mg in tonsil, 5.8 (±0.8) log_10_ RNA copies/mg in reproductive lymph node, and 4.8 (±0.9) log_10_ RNA copies/mg in tracheobronchial lymph node. AUC of IFNα and CCL2 in serum were positively associated with the AUC for viral load in serum (Table 2, Figures 5A and B). As shown in Figure 5, this association was mainly driven by gilts with high cytokine levels and high viral load over time, represented in the upper right quadrant. Importantly, no gilts had high cytokine levels but low viral loads over time (lower right quadrant). A positive association was detected between the AUC for CCL2 in serum and the viral load in lung. IFNα levels in serum trended to be positively associated with viral load in tonsil, while IFNγ levels showed a significant, but negative association (Table 2). No associations could be found for cytokine levels in serum with viral load in tracheobronchial lymph node. Although none of the cytokines measured in supernatants of PRRSv stimulated PBMC were associated with viral load in serum or tissues, levels of IFNγ in supernatants of PMA/Iono stimulated PBMC showed a negative association with viral load in reproductive lymph node (Table 2).

**Table 2:** **Associations between cytokine levels and viral load in serum and tissues following PRRSv infection in pregnant gilts**

**PRRSv RNA concentration (log**_**10**_ **/μL or mg)**	**Serum cytokine levels (AUC)**			**PMA/Iono supernatant (AUC)**
	**IFNα**	**CCL2**	**IFNγ**	**IFNγ**
Serum (AUC)	*P* = 0.003	*P* < 0.001	ns	ns
	β = 0.002134	β = 0.000104		
Lung	ns	*P* = 0.010	ns	ns
		β = 0.000010		
Tonsil	*P* = 0.016	ns	*P* < 0.001	ns
	β = 0.000185		β = −0.000115	
Repro LN	ns	ns	ns	*P* = 0.002
				β = −0.0000004

The associations between cytokine levels over time (represented as area under curve (AUC)) and viral load in gilt serum and tissues are presented. *P*-values (regression coefficients, β) were obtained by univariate, multilevel mixed-effects regression models. Only analytes that were found to be significantly associated with viral load in either serum or any of the tested tissues are included. No association was found among cytokines in supernatants of PRRSv stimulated PBMC and viral load. Repro LN = reproductive lymph node. PMA/Iono supernatant AUC = AUC of IFNγ levels secreted from PMA/Iono stimulated PBMC from PRRSv infected gilts. ns = not significant (*P* > 0.01).

### Relationships between cytokines and fetal mortality rate

In INOC gilts, the percentage of dead fetuses 21 days post inoculation varied from 0% to 94.4% (mean 41.0 ± 22.8%). AUC of IFNα in supernatants of PRRSv stimulated PBMC was significantly associated with percent dead (β = 0.001, *P* = 0.006) (Figure 5C), similarly AUC of IFNα in serum trended to be positively associated with fetal death (β = 0.0035, *P* = 0.060). For cytokine levels in supernatants, a negative trend was detected between the AUC of IL10 in PMA/Iono stimulated PBMC and percent dead fetuses (β = −0.001, *P* = 0.045). For the remaining analytes, levels in serum or in supernatants were not associated with fetal death.

## Discussion

The results presented herein are part of an extensive and complex dataset obtained in a large scale, multi-institutional project aimed at finding phenotypic and genotypic predictors of PRRSv resistance in pregnant gilts. The objectives of this present study were to investigate cytokine responses in serum and supernatants of PBMC, either stimulated with PRRSv or a nonspecific mitogen (PMA/Iono), and to determine possible associations with the outcome of infection in a reproductive model. In order to investigate genotypic variation in the severity of reproductive PRRS, a large number of gilts were experimentally inoculated in the third trimester of gestation, while the number of CTRL gilts was reduced to the minimum needed to provide baseline data.

PMA, a diester of phorbol and potent tumor promoter which activates the signal transduction enzyme protein kinase C, is often used in conjunction with ionomycin, an ionophore inducing calcium transport into the cell, in order to stimulate immune responses in vitro [3,16]. In the present experiment, PMA/Iono stimulation of PBMC induced the secretion of all cytokines tested with the exception of IFNα. Four distinct patterns were observed in the way PRRSv infection altered PBMC cytokine secretions after PMA/Iono stimulation. The altered secretion of cytokines by PMA/Iono stimulated PBMC from INOC gilts after infection indicates a PRRSv immunomodulatory effect. However, the biological meaning of these findings is difficult to interpret. When testing the associations between cytokine levels in supernatants of PMA/Iono stimulated PBMC and viral load or fetal mortality rate, the only significant association was between IFNγ and viral load in reproductive lymph node, where higher levels of IFNγ were associated with a lower viral burden.

Serum IFNγ levels were significantly increased in INOC gilts early in infection. This finding agrees with several reports of either numeric or significant increases in the number of IFNγ secreting cells by enzyme-linked immunospot (ELISPOT) assay, or in the protein levels measured in serum of pigs after PRRSv inoculation [12,17-24]. IFNγ levels in supernatants of PRRSv stimulated PBMC also showed a slight increase on D2, which was not statistically significant. Unlike IFNα and CCL2, no association was found between IFNγ levels in serum and viral load in gilt serum over time. On the other hand, viral loads in tonsil were negatively associated with IFNγ levels in serum indicating a possible protective effect of IFNγ. However, IFNγ levels over time were not associated with fetal death. In contrast, Lowe et al. [25] showed that a strong cell mediated immune response determined by use of an IFNγ ELISPOT was correlated with protection against reproductive PRRS in 3 of 4 investigated farms.

The present study found IFNα to be one of the most relevant cytokines in PRRSv infection. High levels of IFNα were produced by PRRSv stimulated PBMC from CTRL gilts and INOC gilts before inoculation, which is in contrast to previous reports [26,27]. IFNα production, however, was significantly decreased in PBMC collected at all time points following PRRSv inoculation. The levels of IFNα in supernatants of PRRSv stimulated PBMC were positively associated with fetal mortality. Interestingly, it was previously demonstrated that North American type PRRS viruses are potent inhibitors of type 1 interferon production in pDC [28], which are a major source of IFNα and play a key role in the early control of virus replication and the development of adaptive antiviral immune responses [29]. By contrast, a recent study showed that various type 1 and type 2 PRRSv strains induced IFNα secretion by pDC and showed either no or only weak suppression of IFNα secretion [27]. These recent data corroborate our findings of increased levels of IFNα in serum from INOC gilts. However, results of IFNα measurements in serum from PRRSv infected pigs are quite inconsistent. While some studies could not detect significant IFNα increases in serum of PRRSv inoculated pigs within one week of infection [12,18], others did [19,30]. Increased levels of IFNα might have negative effects in regards to PRRSv infection, since it was previously shown that IFNα up-regulated the expression of sialoadhesin and therefore enhanced PRRSv infection of monocytes [31].

In agreement with our results, Souza et al. [32] found significant increases in serum IFNα and CCL2 levels of nursery piglets within the first 2 weeks after inoculation with PRRSv 97–7895, the same virus strain as used for this study. IFNα and CCL2 levels in serum were also associated with PRRSv RNA concentration and growth performance where piglets with low concentration in serum showed a faster return to basal levels of IFNα and CCL2 in serum compared to highly viremic animals. Although we demonstrate the biological importance of INFα in the reproductive PRRSv model herein, the relevance of CCL2 responses in reproductive PRRS is questionable. Although serum CCL2 levels were positively associated with viral burden in serum and lung tissue in the present study, no association was detected between CCL2 levels and fetal death. We propose the role of CCL2 in PRRSv infection primarily involves monocyte recruitment in lung, and is unrelated to mechanisms of fetal death or survival.

Although IL8 levels significantly increased in supernatants of PRRSv stimulated PBMC from INOC gilts after infection, this finding did not have any biological relevance as IL8 levels were not associated with any measured outcome, including PRRSv viral levels or fetal mortality rate. In agreement with previous reports [12,19], no significant increase in serum IL8 levels was detected in INOC compared to CTRL gilts.

In conclusion, PRRSv infection increased IFNα, CCL2, and IFNγ levels in serum but only before 7 dpi. Viral load in gilt serum was positively associated with serum IFNα and CCL2 levels. Although CCL2 and IFNγ were not associated with fetal outcome, IFNα levels in serum trended to be positively associated with fetal mortality rate. The importance of IFNα in regards to PRRSv infection was demonstrated by the significant inhibition of IFNα secretion by PRRSv stimulated PBMC from INOC gilts after infection, and a significant positive association between fetal mortality rate and levels of IFNα in supernatants from PRRSv stimulated PBMC. Therefore, IFNα secretion could be used as a possible indicator of viral load and severity of reproductive PRRS. By contrast, IL8 was not associated with viral load or fetal death even though IL8 secretion by PRRSv stimulated PBMC from INOC gilts was significantly increased at all investigated time points after infection. Finally, levels of IFNγ in supernatants of PMA/Iono stimulated PBMC were suppressed by 21 days post-inoculation, and were negatively associated with viral load in reproductive lymph node. Together with the fact that IFNγ levels in serum were negatively associated with viral load in tonsil, this suggests a protective effect of IFNγ in PRRSv pathogenesis.

## Additional files

Additional file 1:
**Mean cytokine levels (SD) in serum.** Mean cytokine (SD) levels in serum are presented for the 8 analysed cytokines from 111 INOC and 19 CTRL gilts on respective study days post inoculation. If the repeated measures, multilevel mixed-effects regression model demonstrated values in INOC significantly differed from CTRL gilts over all experimental days (DAY*INOC), group differences on individual days were compared (INOC_CTRL). Due to multiple comparisons, *P* < 0.01 was considered statistically significant. ns = not significant. Additional file 2:
**Mean cytokine levels (SD) in supernatants of unstimulated and PRRSv stimulated PBMC.** Mean (SD) cytokine levels in supernatants of unstimulated and PRRSv stimulated PBMC are presented for the 8 analysed cytokines from 111 INOC and 19 CTRL gilts on the respective study days post inoculation. Adjusted values were calculated by subtracting values in supernatants of unstimulated cells from PRRS stimulated cells and used in statistical analyses. Statistics determined whether values in INOC significantly differed from CTRL gilts over all experimental days (DAY*INOC), or on individual days (INOC_CTRL). Due to multiple comparisons, *P* < 0.01 was considered statistically significant; ns = not significant. Additional file 3:
**Mean cytokine levels (SD) in supernatants of unstimulated and PMA/Iono stimulated PBMC.** Mean (SD) cytokine levels in supernatants of unstimulated and phorbol myristate acetate/Ionomycin (PMA/Iono) stimulated PBMC are presented for the 8 analysed cytokines from 111 INOC and 19 CTRL gilts on the respective study days post inoculation. Adjusted values were calculated by subtracting values in supernatants of unstimulated cells from PMA/Iono stimulated cells and used in statistical analyses. Statistics determined whether values in INOC significantly differed from CTRL gilts over all experimental days (DAY*INOC) or on individual days (INOC_CTRL). Due to multiple comparisons, *P* < 0.01 was considered statistically significant. ns = not significant.

## Abbreviations

(Ab): Antibody
(APC): Antigen-presenting cells
(AUC): Area under the curve
(BSA): Bovine serum albumin
(CCL2): Chemokine ligand 2
(CV): Coefficient of variation
(DC): Dendritic cells
(ELISA): Enzyme-linked immunosorbent assay
(ELISPOT): Enzyme-linked immunospot assay
(FBS): Fetal bovine serum
(FMIA): Fluorescent Microsphere Immunoassays
(INOC): Gilts inoculated with PRRSv
(CTRL): Gilts that were sham inoculated
(IFNα): Interferon alpha
(IFNγ): Interferon gamma
(IPCs): Interferon producing cells
(IL): Interleukins
(MHC): Major histocompatibility complex
(MFI): Mean fluorescence intensity
(NK cells): Natural killer cells
(PBMC): Peripheral blood mononuclear cells
(PMA/Iono): Phorbol myristate acetate/Ionomycin
(PBS): Phosphate buffered saline
(PBS-BN): PBS with 0.05% sodium azide
(PBST): PBS +0.05% Tween 20
(pDC): Plasmacytoid dendritic cells
(PRRSv): Porcine Reproductive and Respiratory Syndrome virus
(QC): Quality control
(qRT-PCR): Quantitative reverse transcription polymerase chain reaction
(Th1): T-helper 1
(Th2): T-helper 2
(SAPE): Strepavidin-R-Phycoerythrin
(TNFα): Tumor necrosis factor-alpha
