# Supplementary material for: Cytokine profiles in pregnant gilts experimentally infected with porcine reproductive and respiratory syndrome virus and relationships with viral load and fetal outcome
Source: Vet Res. 2014 Dec 6;45:113. doi: 10.1186/s13567-014-0113-8 (PMC4333882; doi:10.1186/s13567-014-0113-8)
Supplement: Additional file 1: — Mean cytokine levels (SD) in serum. Mean cytokine (SD) levels in serum are presented for the 8 analysed cytokines from 111 INOC and 19 CTRL gilts on respective study days post inoculation. If the repeated measures, multilevel mixed-effects regression model demonstrated values in INOC significantly differed from CTRL gilts over all experimental days (DAY*INOC), group differences on individual days were compared (INOC_CTRL). Due to multiple comparisons, P < 0.01 was considered statistically significant. ns = not significant. [file 13567_2014_113_MOESM1_ESM.docx]

|  |  | **Mean (SD)** | | ***P*-value** | ***P*-value** |
| --- | --- | --- | --- | --- | --- |
| **Analyte** | **day** | **CTRL** | **INOC** | **DAY*INOC** | **INOC_CTRL** |
| IL1β | 0 | 422 (1332) | 60 (208) | ns |  |
|  | 2 | 424 (1372) | 51 (168) |  |  |
|  | 6 | 496 (1419) | 53 (195) |  |  |
|  | 19 | 653 (1714) | 58 (112) |  |  |
| IL8 | 0 | 170 (190) | 113 (117) | ns |  |
|  | 2 | 154 (188) | 104 (105) |  |  |
|  | 6 | 183 (219) | 146 (116) |  |  |
|  | 19 | 265 (360) | 124 (125) |  |  |
| CCL2 | 0 | 1420 (963) | 1051 (557) | < 0.001 | ns |
|  | 2 | 1210 (938) | 6056 (4452) |  | < 0.001 |
|  | 6 | 1349 (1020) | 4198 (2655) |  | < 0.001 |
|  | 19 | 1637 (1225) | 1401 (1079) |  | ns |
| IFNα | 0 | 52 (207) | 10 (37) | < 0.001 | ns |
|  | 2 | 52 (206) | 448 (264) |  | < 0.001 |
|  | 6 | 51 (197) | 80 (59) |  | < 0.001 |
|  | 19 | 80 (321) | 8 (12) |  | ns |
| IFNγ | 0 | 81 (281) | 92 (291) | < 0.001 | ns |
|  | 2 | 84 (286) | 135 (231) |  | < 0.001 |
|  | 6 | 92 (252) | 101 (292) |  | ns |
|  | 19 | 104 (273) | 44 (83) |  | ns |
| IL12 | 0 | 179 (352) | 149 (344) | ns |  |
|  | 2 | 165 (346) | 99 (142) |  |  |
|  | 6 | 155 (405) | 132 (330) |  |  |
|  | 19 | 181 (342) | 287 (734) |  |  |
| IL4 | 0 | 13 (35) | 11 (24) | ns |  |
|  | 2 | 13 (32) | 8 (19) |  |  |
|  | 6 | 14 (37) | 8 (23) |  |  |
|  | 19 | 16 (35) | 23 (75) |  |  |
| IL10 | 0 | 50 (150) | 13 (26) | ns |  |
|  | 2 | 47 (146) | 11 (22) |  |  |
|  | 6 | 55 (149) | 13 (23) |  |  |
|  | 19 | 83 (210) | 24 (45) |  |  |
