# Supplementary material for: Cytokine profiles in pregnant gilts experimentally infected with porcine reproductive and respiratory syndrome virus and relationships with viral load and fetal outcome
Source: Vet Res. 2014 Dec 6;45:113. doi: 10.1186/s13567-014-0113-8 (PMC4333882; doi:10.1186/s13567-014-0113-8)
Supplement: Additional file 2: — Mean cytokine levels (SD) in supernatants of unstimulated and PRRSv stimulated PBMC. Mean (SD) cytokine levels in supernatants of unstimulated and PRRSv stimulated PBMC are presented for the 8 analysed cytokines from 111 INOC and 19 CTRL gilts on the respective study days post inoculation. Adjusted values were calculated by subtracting values in supernatants of unstimulated cells from PRRS stimulated cells and used in statistical analyses. Statistics determined whether values in INOC significantly differed from CTRL gilts over all experimental days (DAY*INOC), or on individual days (INOC_CTRL). Due to multiple comparisons, P < 0.01 was considered statistically significant; ns = not significant. [file 13567_2014_113_MOESM2_ESM.docx]

|  |  | **Mean (SD)** | | | |  |  |
| --- | --- | --- | --- | --- | --- | --- | --- |
|  |  | **CTRL** | | **INOC** | | ***P*-value** | ***P*-value** |
| **Analyte** | **day** | **unstimulated PBMC** | **PRRSv stimulated PBMC** | **unstimulated PBMC** | **PRRSv stimulated PBMC** | **DAY*INOC** | **INOC_CTRL** |
| IL1β | 0 | 62 (115) | 47 (109) | 127 (421) | 49 (101) | ns |  |
|  | 2 | 269 (944) | 90 (248) | 37 (98) | 18 (57) |  |  |
|  | 6 | 114 (264) | 64 (143) | 16 (41) | 13 (51) |  |  |
|  | 19 | 184 (327) | 118 (241) | 58 (178) | 38 (133) |  |  |
| IL8 | 0 | 7527 (8016) | 2534 (2748) | 6778 (8691) | 2205 (2658) | 0.001 | ns |
|  | 2 | 10286 (11475) | 3178 (4635) | 2173 (6030) | 820 (2092) |  | < 0.001 |
|  | 6 | 8028 (7984) | 2421 (2908) | 1617 (3379) | 957 (1747) |  | < 0.001 |
|  | 19 | 10573 (12968) | 2793 (3972) | 5444 (8389) | 2875 (3054) |  | 0.003 |
| CCL2 | 0 | 690255 (1042083) | 273398 (249273) | 943208 (4338070) | 229556 (166435) | ns |  |
|  | 2 | 746879 (1134109) | 334640 (442207) | 180318 (439923) | 179155 (152628) |  |  |
|  | 6 | 696515 (1173464) | 287720 (314639) | 140489 (328987) | 116492 (89733) |  |  |
|  | 19 | 505259 (487587) | 276620 (244705) | 510528 (1352768) | 214204 (199238) |  |  |
| IFNα | 0 | 5 (2) | 1582 (1030) | 7 (13) | 1676 (2086) | < 0.001 | ns |
|  | 2 | 7 (10) | 1685 (1574) | 11 (24) | 939 (984) |  | 0.010 |
|  | 6 | 7 (7) | 1619 (1018) | 7 (7) | 64 (54) |  | < 0.001 |
|  | 19 | 17 (37) | 2567 (2123) | 6 (9) | 203 (217) |  | < 0.001 |
| IFNγ | 0 | 76 (170) | 117 (302) | 269 (947) | 252 (785) | ns |  |
|  | 2 | 108 (240) | 244 (698) | 114 (445) | 452 (3289) |  |  |
|  | 6 | 125 (296) | 204 (456) | 85 (364) | 90 (203) |  |  |
|  | 19 | 121 (301) | 176 (386) | 154 (648) | 234 (431) |  |  |
| IL12 | 0 | 20 (48) | 4 (13) | 22 (47) | 13 (37) | ns |  |
|  | 2 | 14 (41) | 2 (11) | 11 (30) | 4 (19) |  |  |
|  | 6 | 25 (50) | 8 (24) | 29 (51) | 10 (32) |  |  |
|  | 19 | 39 (61) | 8 (29) | 31 (57) | 9 (30) |  |  |
| IL4 | 0 | 7 (7) | 10 (11) | 7 (11) | 9 (18) | ns |  |
|  | 2 | 8 (8) | 11 (12) | 6 (11) | 10 (22) |  |  |
|  | 6 | 8 (9) | 11 (16) | 6 (10) | 8 (16) |  |  |
|  | 19 | 8 (9) | 11 (14) | 5 (10) | 7 (16) |  |  |
| IL10 | 0 | 1 (2) | 0 (0) | 1 (3) | 0 (0) | ns |  |
|  | 2 | 1 (3) | 0 (0) | 0 (1) | 0 (0) |  |  |
|  | 6 | 1 (3) | 0 (0) | 0 (2) | 0 (0) |  |  |
|  | 19 | 0 (0) | 0 (0) | 0 (2) | 0 (0) |  |  |
