# Supplementary material for: Cytokine profiles in pregnant gilts experimentally infected with porcine reproductive and respiratory syndrome virus and relationships with viral load and fetal outcome
Source: Vet Res. 2014 Dec 6;45:113. doi: 10.1186/s13567-014-0113-8 (PMC4333882; doi:10.1186/s13567-014-0113-8)
Supplement: Additional file 3: — Mean cytokine levels (SD) in supernatants of unstimulated and PMA/Iono stimulated PBMC. Mean (SD) cytokine levels in supernatants of unstimulated and phorbol myristate acetate/Ionomycin (PMA/Iono) stimulated PBMC are presented for the 8 analysed cytokines from 111 INOC and 19 CTRL gilts on the respective study days post inoculation. Adjusted values were calculated by subtracting values in supernatants of unstimulated cells from PMA/Iono stimulated cells and used in statistical analyses. Statistics determined whether values in INOC significantly differed from CTRL gilts over all experimental days (DAY*INOC) or on individual days (INOC_CTRL). Due to multiple comparisons, P < 0.01 was considered statistically significant. ns = not significant. [file 13567_2014_113_MOESM3_ESM.docx]

|  |  | **Mean (SD)** | | | |  |  |
| --- | --- | --- | --- | --- | --- | --- | --- |
|  |  | **CTRL** | | **INOC** | | ***P*-value** | ***P*-value** |
| **Analyte** | **day** | **unstimulated PBMC** | **PMA/Iono stimulated PBMC** | **unstimulated PBMC** | **PMA/Iono stimulated PBMC** | **DAY*INOC** | **INOC_CTRL** |
| IL1β | 0 | 48 (79) | 758 (323) | 85 (370) | 695 (313) | < 0.001 | ns |
|  | 2 | 109 (354) | 880 (544) | 15 (35) | 379 (319) |  | < 0.001 |
|  | 6 | 73 (130) | 752 (402) | 13 (39) | 1011 (539) |  | 0.009 |
|  | 19 | 145 (368) | 680 (384) | 41 (137) | 839 (492) |  | 0.022 |
| IL8 | 0 | 3984 (3718) | 26932 (25513) | 4528 (9731) | 24285 (18678) | 0.001 | ns |
|  | 2 | 4862 (6020) | 31128 (31716) | 780 (1540) | 12353 (14991) |  | 0.001 |
|  | 6 | 4504 (5323) | 25814 (24276) | 1148 (2324) | 35135 (93870) |  | ns |
|  | 19 | 4932 (5296) | 27057 (27871) | 3316 (5949) | 25070 (16040) |  | ns |
| CCL2 | 0 | 162263 (227362) | 4790 (3814) | 140129 (281147) | 6077 (12649) | 0.001 | ns |
|  | 2 | 489394 (1569004) | 7073 (9714) | 67762 (109094) | 8768 (19387) |  | 0.002 |
|  | 6 | 218192 (428943) | 4069 (3216) | 60922 (116522) | 9945 (15504) |  | 0.003 |
|  | 19 | 145926 (140194) | 5254 (5207) | 147429 (289303) | 5075 (6436) |  | ns |
| IFNα | 0 | 6 (5) | 7 (5) | 5 (5) | 8 (7) | ns |  |
|  | 2 | 5 (4) | 9 (8) | 6 (5) | 12 (23) |  |  |
|  | 6 | 5 (4) | 8 (5) | 5 (4) | 10 (9) |  |  |
|  | 19 | 5 (4) | 9 (7) | 4 (4) | 7 (7) |  |  |
| IFNγ | 0 | 46 (200) | 44994 (39344) | 74 (421) | 44081 (29634) | 0.039 | ns |
|  | 2 | 80 (349) | 49361 (39376) | 39 (313) | 35971 (25445) |  | ns |
|  | 6 | 565 (2052) | 64233 (108441) | 30 (184) | 42861 (39444) |  | ns |
|  | 19 | 138 (600) | 53751 (33953) | 41 (295) | 38568 (28957) |  | 0.023 |
| IL12 | 0 | 64 (77) | 123 (94) | 53 (74) | 179 (205) | 0.007 | ns |
|  | 2 | 38 (54) | 135 (151) | 39 (60) | 14 (41) |  | < 0.001 |
|  | 6 | 45 (65) | 114 (150) | 66 (74) | 75 (145) |  | ns |
|  | 19 | 35 (53) | 114 (130) | 64 (74) | 103 (211) |  | ns |
| IL4 | 0 | 5 (6) | 221 (129) | 5 (9) | 228 (173) | < 0.001 | ns |
|  | 2 | 5 (6) | 233 (166) | 4 (7) | 252 (218) |  | ns |
|  | 6 | 6 (8) | 222 (140) | 4 (6) | 249 (187) |  | ns |
|  | 19 | 6 (9) | 247 (144) | 4 (8) | 171 (139) |  | 0.006 |
| IL10 | 0 | 0 (2) | 536 (323) | 1 (2) | 412 (244) | < 0.001 | ns |
|  | 2 | 0 (0) | 560 (308) | 0 (1) | 224 (192) |  | < 0.001 |
|  | 6 | 0 (0) | 498 (314) | 0 (1) | 389 (269) |  | ns |
|  | 19 | 0 (0) | 540 (285) | 0 (1) | 440 (320) |  | ns |
